# Exploring participant experiences with the autoimmune protocol (AIP) diet in rheumatoid arthritis: a longitudinal qualitative study

**DOI:** 10.1017/jns.2026.10135

**Published:** 2026-08-03

**Authors:** Julianne McNeill, Gael J. Mearns, Rebecca Grainger, Caryn Zinn

**Affiliations:** 1 AUT Human Potential Research Institute, Faculty of Health and Environmental Sciences, Auckland University of Technology, New Zealand; 2 School of Clinical Sciences, Auckland University of Technology, New Zealand; 3 Department of Medicine, University of Otago Wellington, New Zealand

**Keywords:** adults, AIP diet, autoimmune protocol diet, dietary participant experiences, elimination reintroduction diet, longitudinal qualitative research, one-year follow-up, rheumatoid arthritis

## Abstract

Many dietary interventions have been conducted in rheumatoid arthritis (RA) with quantitative outcomes the key focus; qualitative research exploring participant experiences remains limited. The autoimmune protocol (AIP) diet is an elimination reintroduction approach designed for autoimmune conditions. This study explored the experiences of nine adults with RA who completed an eight-week AIP dietary intervention, with semi-structured interviews conducted immediately post-intervention and again one year later. Interviews explored participants’ motivations, challenges, enablers, and perceived effects of the intervention, their experiences with food reintroduction, and the degree to which dietary changes were sustained. Thematic analysis identified three themes related to initial intervention experiences. **Open to change,** captured initial motivations, trust in the protocol and willingness to attempt substantial dietary change. **Navigating dietary transition** described the mental, practical, and social strategies used to manage challenges and support adherence. **Reflections on benefits and looking ahead**, captured perceived health changes and intentions for future dietary practices. At one-year follow-up, three further themes were identified. **Personalised reintroductions and responses** highlighted how participants observed the effects of foods on RA and health. **Finding and sustaining balance** reflected the ongoing process of negotiating dietary structure with flexibility, particularly in social contexts. **Reflections on wellbeing and future intentions** encompassed perceived benefits, persistent challenges, and plans for ongoing dietary management. High initial adherence was facilitated by participant motivation and structured study support. The reintroduction protocol was perceived as burdensome, however, most participants sustained meaningful dietary change, even without ongoing formal guidance.

## Introduction

Rheumatoid arthritis (RA) is a chronic inflammatory autoimmune disease characterised by synovial joint inflammation, leading to pain, swelling, stiffness, and, if untreated, progressive joint damage. Globally, RA affects approximately 18.5 million people, with prevalence increasing with age. Women are disproportionately affected, with a female-to-male ratio of 2.5:1.^(1)^ In New Zealand an estimated 3.2% of the population, around 133,000 people, are living with RA.^(2)^ RA reduces quality of life (QOL) and increases the risk of co-morbidities arising from both disease progression and pharmacological treatments. These co-morbidities include CVD, certain cancers, osteoporosis, gastrointestinal and respiratory conditions.^(3,4)^

Disease modifying anti-rheumatic drugs remain the cornerstone of modern pharmacological treatment for RA. However, despite advances in drug therapies, many individuals continue to experience persistent symptoms and functional impairment.^(5)^ As a result, people with RA have an interest in exploring dietary strategies to manage symptoms, reduce disease impact, and enhance QOL.^(6)^ Interest in dietary interventions for RA has persisted for over a century, with numerous clinical studies conducted to evaluate their potential to decrease inflammation, address comorbidities, and improve quality of life.^(7)^ More recently, emerging evidence implicating gut microbiome alterations and changes in intestinal permeability in RA pathophysiology has further stimulated interest in diet strategies targeting these mechanisms.^(8,9)^

Several dietary intervention studies in RA cohorts have reported positive short-term outcomes such as reductions in pain and improved QOL measures.^(10,11)^ However, most studies have focused on quantitative outcomes and have rarely examined participants experiences. This represents a notable gap in the literature. Qualitative research can provide valuable insights into participants’ experiences of transitioning to and adapting to a new diet, navigating challenges, and identifying factors that facilitate motivation and adherence to study protocols.

Restrictive dietary interventions, particularly those that exclude entire food groups, and aim to improve dietary quality, can impose a substantial burden on participants, and be difficult to maintain after the study completion. Most RA dietary intervention research has focused on short-term outcomes, with sparse long-term follow-up, either qualitative or quantitative. Evaluating the extent to which participants maintain dietary changes post-intervention, as well as the barriers and facilitators they encounter without structured support, is crucial for understanding the sustainability of such approaches. Therapeutic diets often require ongoing adherence to dietary restrictions, and maintaining these changes in social situations can pose significant challenges.^(12)^

In other contexts, such as weight loss management, sustaining extensive dietary change is generally observed as difficult, with long-term studies typically reporting low adherence and high rates of attrition.^(13)^ Understanding the behavioural dynamics of dietary change in people with RA and examining similarities and differences compared to other populations, is essential to improving the design, effectiveness and sustainability of such interventions.

To date, only three RA dietary interventions have incorporated qualitative methods, with time points ranging from immediately post intervention to five years later. Collectively they highlight the importance of programme structure, education and social support for adherence, and food accessibility, and difficulty in social situations as challenges.^(12,14–16)^

While quantitative measures assess the effectiveness of a dietary intervention, they offer limited insight into how participants engage with dietary change, navigate adherence or perceive the impact of diet on their condition and overall health. Qualitative research is needed to explore the practical, emotional and social processes involved, as well as the influence of study design, on adopting and maintaining dietary changes.^(17)^ Post intervention, qualitative enquiry can elucidate the extent to which dietary changes are maintained and the practical, social and psychological factors that influenced adherence.

This longitudinal qualitative study addresses this gap by exploring participants’ experiences immediately after completing a restrictive dietary intervention for RA and again one year later. It examines how participants navigated and adhered to the autoimmune protocol (AIP) diet during the intervention, and how they managed food reintroductions, and adapted their diets over the following year without ongoing dietary support.

## Methods

### Study design

The qualitative research reported here was part of a two-phase dietary intervention study conducted in 2021 that evaluated the effectiveness of the AIP diet in people with RA.^(18)^ Quantitative outcomes (reported elsewhere) included self-reported disease activity and impact, using the Routine Assessment of Patient Index Data 3 (RAPID3) and Rheumatoid Arthritis Impact of Disease patient-reported outcome questionnaires, biometric measurements, and nutritional analysis.^(18)^

For this study, a qualitative descriptive design was adopted, consistent with a naturalistic paradigm^(19,20)^ to capture participants’ experiences of the AIP diet, including the challenges of maintaining adherence and of transitioning away from this highly restrictive dietary pattern. Data were collected through individual semi-structured interviews and inductively analysed using Braun & Clarke’s reflexive thematic analysis.^(21–23)^ This combination allowed us to remain close to the data while also providing an interpretive synthesis of participants’ experiences of adopting and sustaining the AIP diet.

### Intervention and follow-up

The intervention study was conducted between June and November 2021, with remote delivery and support during the final three months due to COVID-19 restrictions. The study followed a within-subject design: Control phase (weeks 0–4), where participants consumed their habitual diet, and an intervention phase (weeks 5–12), where participants adhered to a self-prepared ad-libitum AIP diet. Following completion of the 12-week study, participants received advice on staged food reintroductions based on AIP principles,^(24)^ but no further formal support. The reintroduction process involved introducing each food individually over 2–3 days while monitoring for symptom recurrence; foods causing symptom exacerbation were excluded, while others were retained. Participants were neither required nor expected to continue the diet, and at the time were unaware of any follow-up study.

One year later (October–November 2022), all nine participants who had completed the AIP pilot study were invited to participate in repeat data collection and interviews; all agreed to take part.

### Participants

Participants were recruited from Auckland, New Zealand between May and August 2021, through social media platforms and Arthritis New Zealand. Inclusion criteria were adults aged 18 years or over, with a clinically confirmed RA diagnosis for at least six months, stable use of all medications and supplements for eight weeks, fluency in English, computer literacy, access to a computer and smartphone, and the ability to attend four in-person visits at Auckland University of Technology (AUT) across the 12-week study period.

Exclusion criteria were pregnancy, health conditions requiring specific dietary management (e.g., diabetes mellitus, renal impairment, liver disease) or current adherence to a diet excluding any food group. Eligible participants received detailed study information, including details of procedures, responsibilities, and consent implications. Participants commenced the study immediately upon recruitment, continued their standard medical care and maintained any current supplements.

### The intervention diet and adherence support

The AIP diet followed guidelines from Ballantyne,^(25)^ and the AIP Certified Coach programme.^(26)^ Excluded food included grains, pseudograins, legumes, dairy products, nightshade vegetables and fruit, seeds, nuts, eggs, alcohol, sugar, food additives (e.g., synthetic colours, flavours, emulsifiers, and preservatives), and all processed foods containing additives.^(25,27)^ Permitted foods included unprocessed meat, poultry, seafood, all fruits and vegetables except nightshades, and fats such as avocado, olive, coconut oils, and animal fats. Alternative non-grain flours were allowed (e.g., cassava, arrowroot, tigernut, green banana, coconut). Participants were encouraged to consume omega-3-rich seafood, a variety of fruits and vegetables, organ meats, and fermented foods. No food was provided; participants purchased their own ingredients and prepared meals independently and ate to satiety.

At the end of the control phase, participants received verbal and written instructions from the researcher, a registered nutritionist (JM) including detailed guidelines, food lists, recipes, shopping resources, and strategies for overcoming adherence challenges. Ongoing support was available via phone or email, and peer support was offered through a moderated private Facebook group. Participants recorded any non-compliant foods on a daily checklist submitted weekly.

### Qualitative data collection

In-depth one-on-one interviews with participants were conducted by the study researcher, (JM) via Zoom (Zoom Video Communications, Inc.). The interviews followed a semi-structured questionnaire guide developed with input from GM and CZ (Supplementary Tables S1 and S2) and were conducted with all nine participants at two time points. At post-intervention (Week 12), topics included adaptation to the diet, challenges and facilitators of adherence, cost and ingredient availability, perceived health changes, and overall study experience. At one-year follow-up, topics included experiences with food reintroduction, identification of symptom-triggering foods, extent of dietary maintenance, and barriers and facilitators to diet adherence.

Interviews were audio-recorded and transcribed verbatim using Otter.ai, with manual corrections to ensure accuracy and inclusion of non-verbal cues. Each interview lasted from 40 to 80 minutes.

### Data analysis

Interview transcripts were imported into NVivo-12 (Lumivero) for inductive thematic analysis, using Braun and Clarke’s process.^(21,22,28)^ The analysis process included the following steps: 1. Data familiarisation through listening to the interviews, transcript checking, correcting and adding non-verbal cues, and re-reading, noting reflective thoughts. 2. In-text coding from raw data, and generating initial codes in NVivo, with text sections collated in each code. 3. Collation of related codes into subthemes, followed by grouping of subthemes into preliminary themes. 4. Reviewing and refining of themes, subthemes and codes through discussion with co-authors (CZ, GM). This was an iterative process where subthemes and themes were reviewed and rearranged and renamed. Final definition of themes, with subthemes and representative participant quotes were tabulated.

### Reflexivity

Throughout the analytic process, the researcher maintained reflexive awareness of her dual role as both the facilitator of the dietary intervention and qualitative analyst. Codes were developed inductively from the data, with attention paid to the research questions and to capturing participants’ authentic experiences of participating in the study and navigating a restrictive diet. The researcher acknowledged that her prior investment in the AIP diet and close involvement with participants could introduce bias, particularly a tendency to emphasise positive outcomes or overlook critical reflections. To actively mitigate positive bias, interview questions were balanced to elicit both positive and negative experiences (e.g., questions explicitly addressing difficulties, dislikes, cravings, adverse effects, and suggestions for improvements) (Supplementary Tables 1 and 2). Follow-up prompts were used to explore these experiences in greater depth, while the researcher remained mindful of maintaining a neutral interviewing stance. She remained conscious of the knowledge-based asymmetry, that may have led participants to underreport challenges or give more favourable feedback, whether consciously or unconsciously. This reflexive stance aimed to ensure that both favourable and unfavourable experiences were given equal consideration, and that the analysis remained grounded in participants’ voices rather than researcher preconceptions.

### Trustworthiness of the research findings

Trustworthiness of the research requires that findings are credible, dependable and meaningful for participants and the wider research community.^(29)^ This was addressed through the criteria of credibility, transferability, dependability and confirmability.^(30,31)^

Credibility was strengthened through meaningful engagement with participants during the interviews and with interview data during analysis, ensuring that findings reflected participants’ voices and experiences. Transferability was enabled through detailed descriptions of participants, the dietary intervention, and research context to allow readers or other researchers to assess the applicability of findings to other RA populations or dietary interventions. Dependability was supported by maintaining a comprehensive audit trail of the research process, documenting each stage from interview collection through to final thematic decisions.

The data, initial coding, groupings of codes, and the development of subthemes and themes were discussed and reviewed iteratively and collaboratively with co-authors at each stage supporting the confirmability of findings. The primary researcher engaged in reflexivity to remain aware of how personal biases or perspectives could influence the research process. Supplementary tables (Supplementary data tables S2–S8) with coded quotes further demonstrate that the findings are supported by representative data and provide transparency in how interpretations are reached.

To summarise, the supplementary materials provide additional supporting data and methodological detail: Supplementary Tables S1 and S2 contain interview guides for both time points. Supplementary Tables S3–S5 present coded excerpts supporting post-intervention theme development, and Tables S6–S8 the corresponding one-year follow-up data.

## Results

Eleven participants enrolled in the study, and nine started and completed the AIP dietary intervention. Two participants (P8 and 9) withdrew at week four of the habitual diet phase and did not start the AIP diet intervention phase, one due to a recently broken ankle and one who was unable to prepare her own food due changed living circumstances in the COVD-19 lockdown. Participants included seven females (Participants (P) 1, 2, 4, 6, 7, 10, 11) and two males (P3 and 5), mean age 52 years (range 40–63). Participants had a range of living situations, living alone (P7), living with a partner (P5 and 6), solo parent (P4), and two parent families with children (school age to adults, P1, 2, 3, 10 and 11). Much of the study took part during pandemic restrictions, where six people stayed home, and three who were essential workers continued to work outside the home (P3, 5 and 7). Most pandemic lockdown restrictions were lifted at the end of 2021, so that most of the in the year following the pilot study completion participants were free to move outside the home. Participants 8 and 9 who withdrew pre-intervention are not included.

## Qualitative analysis

### Post-study interviews

The three themes identified reflect the stages participants progressed through during the study. The first theme **Open to change,** encompassed participants’ initial motivations for enrolling. The second theme, **Navigating dietary transition,** captured the mental and physical strategies used to adopt the diet, the ways challenges were addressed, and the enablers that supported adherence. The third theme, **Reflections on benefits and looking ahead,** outlined participants’ future dietary intentions.

#### Theme 1: open to change

Participants demonstrated a high level of adherence to the AIP protocol over the eight-week intervention, as evidenced by weekly self-reported checklist submissions. The strong adherence appeared to be shaped by participants’ initial motivations for enrolling, their trust in the researcher and the protocol, and their belief about the potential role of diet in managing autoimmune disease.

##### Subtheme: motivation to change

Participants expressed a willingness to make a substantial change to their diet, viewing the study as a timely opportunity. For some it aligned with pre-existing intentions and dietary philosophy:

*“I’d had a note to sort of look into it (AIP) and find out more about it. So then when I saw your post, I thought, well, this is the perfect opportunity”* (P1).

Others were motivated by a desire to gain control over their eating habits:

*“There was no motivation, really for anything else except to benefit myself to get me on the track to get a decent diet going”* (P2).

Some had previously experienced benefits from dietary changes and saw this as a chance to revisit this, while others hoped the intervention might help manage their RA symptoms:

*“if this was something that could help me decrease my pain meds for RA, then, you know, it was something that I thought was exciting to try”* (P4).

Altruistic motivations, and a desire to contribute to research were also expressed:

*“If it makes a difference to other people, then that’s awesome. Yeah, it’s good to be involved in something like this”* (P4)

##### Subtheme: trust in guidance and protocol

The structured nature of the study and the opportunity to receive guidance from a qualified nutritionist fostered trust for participants, many of whom had experienced dietary advice as confusing or contradictory:

*“It’s so good to have a qualified dietitian to guide you and to push you on the right track and to help you through, whereas when you’re on your own you just don’t have, I didn’t know what to do”* (P2)

Several participants also expressed confidence in the protocol, perceiving it as distinct from typical weight loss diets, and instead focused on addressing autoimmune disease. The scientific rational for the AIP diet was described as logical.

*“And this is a completely different diet, it’s a diet about autoimmune. It’s about your immune system. It’s about getting yourself better.”* (P2)

##### Subtheme: curiosity and hope about a different approach

Hope, curiosity, and open mindedness were commonly expressed as reasons for both enrolling in the study and adhering to the protocol. While participants were generally cautious in their expectations, they expressed hope and were interested in whether dietary change would offer symptom relief.

*“I wouldn’t say any expectations. I was just, you know, really open minded … I was just really curious to know if it would work or have, you know, any effects… just really intrigued to see whether, you know, diet would help with pain and inflammation.”* (P11)

#### Theme 2: navigating dietary transition

The second theme captures participants’ experiences of transitioning to, and maintaining the AIP diet, which required substantial change and the exclusion of many commonly consumed foods.

##### Subtheme: working around the realities of diet transition

The early stage of the intervention demanded substantial mental, emotional and physical preparation. Many participants anticipated feelings of deprivation, hunger, and the loss of familiar enjoyable foods. Some expressed emotional resistance and doubts about their ability to adhere, with a few reporting overeating favourite foods in anticipation of restriction.

**Mental preparation** included “psyching themselves up” and committing to *“100%”* adherence. **Practical preparation** included researching the AIP protocol, planning meals, and collating recipes to establish a sense of control. Strategies varied, some adopted a minimalist approach to reduce stress and focused on simple meals, while others invested effort in sourcing ingredients and meal prepping. Environmental changes such as removing non-compliant foods, marking excluded food items, and posting food lists were also common.

**The initial transition** was experienced as *“completely foreign to begin with”* and cognitively demanding, constantly *“thinking about what, what am I going to cook, you know, looking up recipes and all that”* (P11). The physical load also increased *“So you basically go from cooking one meal a day to three meals a day so yeah, there was definitely preparation there.”* (P5 partner)

Some participants experienced short-term side effects, including hunger, irritability, digestive changes, headaches, and what they perceived as withdrawal-like symptoms from sugar or processed foods.

##### Subtheme: managing challenges to sustain adherence

**The exclusion of convenience foods** meant that time-intensive meal preparations were the most common persistent challenge. To manage this, participants adopted various strategies, including batch cooking and freezing meals, or simplifying meals by using only familiar foods. Some noted that lockdown conditions provided more time, easing this burden.

**Unfamiliar ingredients and new recipes** required experimentation, with variable success. Participants appeared to take these difficulties in their stride:

*“I still haven’t managed to make bread. I made a couple of cricket balls you could kill at 80 yards with…so I just gave up on the bread”* (P2).

Some meals were described as unpalatable or bland, and certain flavours, such as spicy foods, were missed. Cravings, often for sweets, were managed with creative alternatives, such as dried fruit, coconut chips or olives. One participant sought permission to include dairy free dark chocolate to reduce sugar cravings. Temptations were managed through internal motivation, fear of symptom recurrence, commitment to the study, and focus on positive benefits:

*“I think there’s always that little thought in the back of my head because I see a bag of chips and I’m like Oh, I do miss the taste of those, and I smell the coffee, and I Oh, I miss the taste of that. But the willpower to not do it wins. Yeah, so there’s always that little thought, but the gains and all the goodness are stronger.”* (P7)

*“You have those moments where you think…icecream will be nice … and if we weren’t on the study, then I’m damn sure I would have had a couple.”* (P5)

**Social eating** outside the home required navigation; concerns about ingredient transparency and the need to request modifications created discomfort:

*“I would have felt like I was being a bit of a hassle”* (P1).

However, COVID-19 lockdowns limited most social eating to small family or neighbourhood gatherings, where participants managed dietary needs more easily:

*“we’ve had outdoor things with our neighbours over the last few weeks. And I’ve just taken along my own dinner and my soda water every time” (*P11)

Around weeks five- or six, some experienced **“diet fatigue”** and began “counting down” to the end of the intervention. The fixed timeframe however, provided a clear endpoint that helped sustain adherence.

**Costs** were perceived as higher initially due to purchasing AIP compliant ingredients but stabilised over time. Some participants avoided baking to reduce complexity and expense, while others noted the inconvenience of sourcing specialty items, often requiring multiple online suppliers.

##### Subtheme: enablers of adherence

Multiple factors supported adherence, including study structure, social and household support, practical resources, peer connections and the development of new routines.

**External accountability** from the clinical trial format was a strong motivator, creating a desire in participants “do it properly,” out of a commitment to both personal goals and the research. This accountability stopped them giving in to cravings and stress eating during the COVID-19 lockdowns. However, minor lapses or even the thought of “cheating” sometimes invoked guilt, highlighting the internalised pressure to comply.

*“I’m on a clinical trial. I need to follow this’. I wanted to do it as best as I could Yeah, because I didn’t want to, I don’t like letting people down and you don’t get the full benefits if you don’t do it correctly.”* (P7)

*“having somebody there like, you know yourself, who’s been checking in and, you know, reporting to as such every week. It’s like, I can’t cheat! I don’t know. It’s all mindset, I think. …I’d feel really guilty if I had something. I would you know, and then I think I’d punish myself”* (P11)

**Social and household support** was considered essential. Support included encouragement, help with cooking, adapting meals for participants, and reminders to stay on track. Some had adult children or a partner sharing the same meals:

*“definitely my husband doing it as well. You know, being that support person…I just think having somebody eat the same foods as you doesn’t make you feel so isolated.”* (P11),

Another had a friend who gave emotional support:

*“And she goes ‘youre doing so great, but I always knew you’d do great’… it just it’s nice having someone to share it with”* (P7, living solo).

For male participants, female partners managed all the meal planning and cooking, along with sharing the diet. Humour also played a role in encouragement:

“*we’d say ‘Oh, would you like a gin?’ Which meant the soda. (laugh) ‘Oh, yes, I’d love a gin thank you’ (laugh)”* (P5, partner).

**Peer connection** via the private Facebook group was perceived as valuable for sharing recipes, tips and resources, as well as the journey of the AIP diet and having RA. However, one participant noted they might have benefited from initially meeting in person, and another, who did not use Facebook, missed this support.

**Researcher support.** Timely responses to questions, clear guidance, and access to curated materials (e.g., recipes, food lists) were frequently cited as an important facilitator. Participants also accessed AIP information via websites and books for additional recipes and advice

**Emotional and physiological factors** made adherence over time easier. Over time, participants reported that the AIP diet “became easier” and “more normal”. Familiarity with recipes reduced the cognitive and time demands reinforcing sustainability. Some AIP recipes became new favourites: *“I made a butternut lasagna. Yeah, it’s delicious. And we wouldn’t stop eating that now.”* (P3, wife). Participants also observed **reduced cravings** *“I haven’t craved chocolate at all believe it or not, I don’t know why”* (P2) and **greater satiety**: *“I mean, previously, after work, I’d come home three o’clock. You know, and I’d binge on whatever before because I was just so hungry… Whereas now? I don’t really have that.”* (P11).

#### Theme 3: reflecting on benefits and looking ahead

Seven of the nine participants reported experiencing a range of health improvements during the AIP intervention. Participant’s experience of how AIP affected them influenced their intentions for dietary management beyond the intervention period.

##### Subtheme: perceived health changes

Several participants reported improvements in RA related symptoms including **reductions in joint pain, swelling, and stiffness**, describing this benefit as significant, enabling greater physical activity.

*“virtually no joint pain in my hands now which is fantastic…I am aware of pain in one foot but it’s nowhere near as bad as what it used to be, like I’d feel that within 3 Ks sort of thing. But now I can easily walk you know, 8, 9 Ks…there’s definitely less inflammation than I’ve EVER had.”* (P11)

**Increased energy levels**, particularly in the afternoon, and **improved sleep quality** were also noted:

*“I was definitely able to sleep better, I usually wake up with pain and have to stretch a certain way. There’s not the pain. So, I haven’t been having to do that.”* (P4)

**Improvements in other areas** included reductions in digestive symptoms, such as bloating, constipation, diarrhoea, or indigestion, fewer headaches and skin improvements:

*“…had adult acne which is really bad and wouldn’t go away - cleared up.”* (P7).

One post-menopausal participant observed a marked reduction in hot flashes

*“I was having hot flashes, probably three, four times a day. I think if I’ve had four hot flashes since I’ve been on the diet”* (P2).

These benefits were meaningful to participants and reinforced satisfaction with the diet.

**However, not all experiences were positive.** Two participants reported adverse changes. One (P10) described worsening fatigue, increased RA symptoms, weight loss, and reduced appetite, which persisted throughout the intervention. Although gastrointestinal symptoms improved, the overall impact on wellbeing was negative. Another participant (P3) reported no change in RA symptoms and persistent diarrhoea during the later weeks of the diet.

##### Subtheme: future dietary intentions

At the study conclusion, participants reflected on how they intended to approach their diet going forward. Most wished to preserve the benefits they had experienced, and described plans to reintroduce foods gradually, and maintain a more flexible AIP approach rather than strict adherence:

*“I’d like to keep as much of the way I’m eating now, as part of my life, daily life. At the same time, I don’t want to restrict myself if I am going out for dinner or what have you. I don’t want to say no, I can’t have that”* (P4).

One participant, 7, who experienced a substantial improvement in symptoms (a 4.5/10 reduction in RAPID3 score), expressed a commitment to continue the strict AIP protocol for several more weeks before beginning reintroductions.

In contrast, another participant voiced concern about the potential to revert to old habits once the structure of the study ended, highlighting the psychological challenges of sustaining long-term behaviour change without external accountability:

*“it’s so easy to say, well, I’ve done my bit for eight weeks, and I know I feel good. Of course, as soon as you feel good, you start stuffing yourself full of rubbish again.”* (P2).

## One year follow up interviews

Over the year following the intervention, participants’ journeys continued to evolve, with three further themes identified. The first theme, **Personalised re-introductions and responses,** captures the range of strategies used to reintroduce foods in the absence of external structure, along with participants’ observations of effects of those foods. The second theme **Finding and sustaining balance,** describes how participants refined their diets, working out the degree of dietary structure required to preserve health benefits. The final theme**, Reflections on wellbeing and future intentions,** encompassed participants’ assessments of their health over the previous year, the benefits that persisted or diminished, and the dietary approaches they intended to adopt going forward.

### Theme 1: personalised re-introductions and responses

#### Subtheme: approaches to reintroductions

Most participants began reintroducing foods soon after the intervention ended. Strategies ranged from spaced single foods or food groups to a more spontaneous approach, observing the effects of non-AIP foods as they were consumed. Frequently desired foods, such as peanut butter, gluten free bread, eggs, and coffee, were often reintroduced first.

While some initially attempted a structured protocol, several reported losing momentum after a few weeks, describing the process as “too hard” or “boring”:

*“it was hard to, like, reintroduce one thing at a time I found”* (P1).

Only one person (P7) maintained strict AIP for 3.5 months, before following the step-by-step reintroduction process outlined by Ballantyne.^(24)^ In contrast, participant 10 who experienced excessive weight loss during the intervention returned immediately to unrestricted eating to regain weight and restore appetite.

#### Subtheme: responses to reintroduced foods

Some participants observed a return of symptoms reaction after reintroducing certain foods. Two reported reactions to nightshades, particularly potatoes:

*“White potato I had my joints were aching all over, headache and it took about a day before it settled down again”* (P2)

Two others experienced symptoms after consuming a spicy restaurant meal:

*“We went out for a meal and it was the first time I’d had a curry, and my joints were sore. … we went to bed and then I woke up the next day and I was really quite sore.”* (P11).

One participant observed a symptom flare within an hour of eating, while others noticed symptoms the following day, waking up with more inflammation.

Not all participants experienced RA symptom flares, for example:

*“we probably did about six weeks reasonably conscientiously bringing groups in and with no sort of real adverse effects”* (P5)

Some foods were linked to non-RA symptoms. Bread, wheat and dairy were most associated with digestive discomfort (e.g., bloating, diarrhoea, “*glugginess*”), while high glycaemic foods were linked to migraine in one person.

Many foods were reintroduced without perceived negative reactions, leading participants to confidently include them in their diet. Commonly well tolerated foods were eggs, nuts, seeds, coffee, moderate alcohol, chocolate, and gluten free grains.

### Theme 2: finding and sustaining balance

#### Subtheme: seeking balance

Within a few weeks post-intervention, most participants had moved away from strict AIP. They cited the mental effort, cognitive load, and physical demands of maintaining the restrictive protocol: *“I found doing the diet was really restrictive, and it was, like taking up a lot of my brain matter”.* (P4). The successful reintroduction of certain foods made their diets more enjoyable, less restrictive and free from perceived negative effects. Most described their current eating patterns as healthier than their pre-study diet, but more flexible than AIP:

*“on the spectrum, we’re kind of we’re not down the AIP end, but we’re certainly not back where we were. I don’t know. We’re probably somewhere in the middle, you know, pretty considered and conscious about what we eat. We’ll have a bit of alcohol, we’ll have some chocolate, we’ll have some coffee. Those sorts of things in our lives don’t seem to be, don’t think they’re impacting.”* (P5)

#### Subtheme: retaining AIP inspired habits

While some claimed to have reverted to pre-study eating patterns, further discussion revealed that most had retained AIP-inspired principles or habits. These changes: nutrient dense meals, more plant food variety, and avoiding or limiting foods associated with symptoms flares, weren’t experienced as being restrictive:

*“I didn’t want to be under that regime and stress and having to double think what I could eat…, I decided that’s it. I’ve done it. I’m going back to how I eat normally.”… “I guess I’ve pretty much eliminated dairy from my diet. I’m mindful about how much gluten I eat, but not to the point where I don’t have it.”… “I do have a lot more anti -inflammatory food products in my diet, like a protein shake with the blueberries and beetroot and stuff like that. So, I have incorporated them into my daily routine, like breakfast. So that has changed. That’s a regular thing for me now.”* (P4)

Foods most often limited or avoided included gluten, dairy, nightshades, alcohol, and processed foods.

*“For me now, the maintenance seems to be staying away from gluten as much as I can.”* (P5)

*“I’m pretty clear that if I went back to having nightshades at the level that I was, I would have a higher level of pain”* (P6)

*“I haven’t gone completely back to old ways. Still mostly dairy free. Don’t eat a lot of bread…(Alcohol) definitely nowhere near as what we were before”* (P11)

#### Subtheme: maintaining everyday habits

At home, participants found it relatively easy to maintain their chosen dietary patterns. Strategies established during the intervention, such as weekend meal preparation, modifying food delivery kits, batch cooking, and taking home prepared lunches to work, were sustained. Family support remained important, with partners continuing to share meals and family members assisting with meal preparation.

As during the AIP intervention, a gendered dynamic persisted; both male participants reported their female partners took the lead on meal planning and cooking.

**Flexibility was considered important when eating socially.** Participants aimed to make choices aligned with their preferences, such as avoiding gluten, dairy or nightshades, but they didn’t stress if these foods were eaten occasionally:

*“if I do go out, I just look for the best option of what’s on the menu… You know, there might be an ingredient in it that wouldn’t have been allowed on the AIP, but that doesn’t bother me anymore.”* (P1)

“*But if I’m out somewhere, if people ask me, ‘Is there anything you don’t want?’ I’ll say, ‘no nightshades, if possible’. But if I’m served food with nightshades on it’s, it’s an occasional thing.”* (P6)

When travelling, participants employed proactive strategies including bringing their own food, choosing self-catering accommodation, or requesting dietary modifications from hosts or caterers. These approaches enabled participants to maintain dietary control and were not perceived as burdensome.

One participant was unable to sustain dietary habits. She reported a complete return to pre-study eating habits. This participant (P2) had expressed concern about maintaining discipline at the end of the study, and despite beginning with a conscientious approach, eventually stopped trying.

### Theme 3: reflections on wellbeing and future intentions

#### Subtheme: sustained symptom reduction

Six participants said they continued to experience stable or improved RA symptoms over the year, compared to pre-intervention, with lower pain and inflammation levels, and in conjunction with medication, effective disease management. For some, the AIP intervention was described as a turning point or ‘reset’ for their health. These benefits appeared to motivate continued adherence to dietary practices:

*“I think that study was kind of a shifting point for me…. I really don’t get swelling, I sort of don’t know whether it’s the drugs or the diet or a combination of both, but I’m feeling pretty good, best, best I have in years really.”* (P5)

*“one of the reasons I wanted to do the study was we talked about it being like a reset of the system. And I think to some extent, that may have happened because as I say, I definitely have had a pretty pain free year.”* (P6)

#### Subtheme: general wellbeing changes

Beyond RA-specific symptoms, participants described ongoing improvements in general wellbeing. These included improved sleep quality, sustained gastrointestinal health benefits (e.g., reduced bloating, regular bowel movements, absence of indigestion) and a sustained sense of vitality. However, some noted that energy levels had decreased compared to when following strict AIP.

The one participant (P2) who reverted to her pre-study diet, reported this coincided with a return of symptoms, including IBS, menopausal hot flashes, joint pain, and weight regain.

#### Subtheme: persistent health challenges in AIP diet non-responders

Two participants reported worsening clinician-assessed disease activity over the follow-up year. One (P3) experienced persistent diarrhoea, despite medical investigation, as well as increased RA disease activity. The other (P10) struggled with delayed recovery of weight loss and fatigue, requiring input from a nutritionist. Despite increasing her weight and strength, RA disease activity continued to worsen.

#### Subtheme: future dietary intentions

Plans for future eating patterns appeared to be linked to current assessments of wellbeing. Those who noticed a drop in energy compared to strict AIP, or perceived their diet had become less healthy, expressed a desire to return to a stricter version of their current diet.

*“I’m gonna make a good go of it again. But it’s been very difficult mentally in the last last couple of months, in the last year, acknowledging and realising exactly what damage I’ve done.”* (P2, who reverted to pre AIP diet)

*“I know that I’m going to make changes… having salads on a nightly basis or like the roast veggies, making those up again for the week,… getting rid of my wheat not having breads. making things from scratch… but keeping that simple as well.”* (P4)

Others were content to continue their current, balanced regime, maintaining a focus on variety and healthy eating, without sacrificing flexibility or enjoyment:

*“try and sort of keep with the same philosophy as it were, keep with the philosophy of eating as much variety as I can. As much fresh fruit and veg, try and keep with the variety of vegetables… I feel quite well and quite happy. I wouldn’t want to cut out those things that I do enjoy, like my peanut butter toast.”* (P1)

## Discussion

This qualitative study explored nine participants’ experiences of taking part in the AIP diet study, both immediately post intervention, and again at one-year follow-up. Understanding participants’ experiences of navigating the process of changing to and maintaining adherence, is essential to inform the design, delivery and support structures of future dietary studies. Insights into how individuals adapt such diets to everyday life, once outside the supportive study structure, provides valuable understanding of the challenges encountered and the strategies used to sustain long-term dietary change.

It is important to note that the symptom changes described by participants represent their perceptions and experiences of dietary change and should not be interpreted as evidence of clinical efficacy. Rather, these findings provide insight into how participants experienced the intervention and interpreted any changes they observed, which are valuable for understanding and providing context for motivation, adherence, and long-term dietary behaviours.

The primary motivations for participation were hope and curiosity about the potential effects of the diet on RA symptoms, as well as the opportunity to implement dietary change guided by a qualified nutrition professional. These findings align with the 12-week MedRA trial, where participants expressed interest in alternatives to medication, healthier eating patterns, and participation in evidence-based research.^(15)^ Altruistic motives were also expressed, including a desire to contribute to scientific knowledge and benefit others. Comparable motivations have been reported in dietary interventions following prostate cancer radiotherapy, where commitment to study goals served as a key driver.^(32,33)^ A unique motivator in this study was the autoimmune disease-specific relevance of the AIP diet, which participants perceived as more compelling than general healthy eating advice. This interest in disease-specific dietary protocols is consistent with surveys in RA populations, where 12% to 61% of individuals believe that diet influences their condition, and pursue specific diets or alternative therapies in the hope of improvement.^(34)^ Several participants also commented that the scientific rationale for the AIP diet “made sense”, a recognised facilitator of dietary adherence.^(32)^

Recent dietary interventions in RA have primarily focused on dietary quality-based approaches, such as plant-based (vegan) diets^(35,36)^ or Mediterranean diets.^(37,38)^. In contrast, the AIP diet uses broad, multi-category food exclusions based on the premise that certain foods may contribute to symptoms. Unlike other exclusionary approaches, such as vegan diets which exclude food groups indefinitely, AIP includes a structured reintroduction phase designed to identify individual food tolerances, rather than maintain permanent restrictions. The combination of extensive food exclusions and individualised reintroductions distinguishes the AIP experience from other dietary approaches studied in RA. While the elimination phase increases both the cognitive and practical burden of implementation, the opportunity to identify personal symptom-triggering foods may also have contributed to the strong motivation observed among participants in the present study.

### Transitioning to AIP

Transitioning to AIP required substantial cognitive and practical preparation. This dual burden is seldom explored in depth but mirrors findings from other RA dietary studies that include exclusions and substantial change. In the MedRA study, participants reported that sourcing appropriate foods, and the time required for meal planning and cooking affected adherence.^(37)^ The Plants for Joints (PFJ) study identified time required for meal preparation and difficulties eating out as significant barriers.^(14)^ A qualitative study of the Specific Carbohydrate Diet (SCD) in children with juvenile idiopathic arthritis (JIA), which, like AIP, involves strict exclusions and cooking from scratch, identified similar challenges. Barriers included time, cost, sourcing unfamiliar ingredients, the need for meal planning and label reading. In both SCD and AIP, strategies such as removing non-compliant foods, adapting recipes, and batch cooking supported adherence.^(39)^ Across the AIP, MedRA, PFJ, and SCD studies, time constraints were consistently reported as barriers, while meal planning and preparation were identified as key enablers.^(14,15,39)^ Unlike the MedRA study where chronic pain hindered food preparation,^(15)^ only one AIP participant cited this as a barrier. Others reported that preparing simple meals with familiar ingredients helped reduce both the mental and physical burden. Social support emerged as a critical enabler of adherence in the AIP study. Several participants had partners or family members who either adopted the diet themselves or provided practical assistance with shopping and meal preparation. This finding is consistent with evidence that dietary interventions are more effective when a spouse or partner is actively involved.^(40)^ A gendered pattern in dietary implementation was also observed. Female participants often balanced dietary responsibilities alongside caregiving and employment, sometimes receiving help from older children or partners. In contrast the two male participants, both employed full-time, relied heavily on their partners for meal planning and preparation. Similar gender dynamics have been described in dietary interventions of males undergoing prostate cancer radiotherapy, where wives took on most of the responsibility for meal planning and cooking.^(33)^ Support from the researcher was also highly valued, particularly the provision of education, resources, recipes, and timely responses to questions. Similarly, the dietitian’s support in MedRA, and both theoretical and practical guidance in PFJ were viewed as vital for feasibility.^(14,15)^

The COVID-19 lockdowns, which coincided with much of the study, enabled more time for meal preparation and reduced the pressures of social eating. Similar effects were observed in the MedRA study.^(15)^ Social eating with dietary restrictions is typically challenging.^(41)^ Several participants noted that the study structure prevented mindless or unhealthy eating, contrasting with national trends showing increased processed food consumption during lockdown in New Zealand.^(42)^ However, sourcing specialty items and relying on others for grocery shopping were noted difficulties. For essential workers, preparing meals in advance for workdays was crucial.

Peer connection via a private Facebook group fostered a sense of shared experience in both managing RA and adhering to the diet. By contrast, MedRA participants lacked such peer support and viewed this as a disadvantage.^(15)^ The PFJ study incorporated group support (online or in-person), which participants found helpful, although some felt online delivery limited interpersonal connection,^(14)^ a sentiment echoed by several AIP participants. Remote delivery of support, introduced partway through the AIP study, also revealed limitations. One participant’s significant weight loss went underreported during online sessions, an issue that may have been identified with in-person contact.

### Long term behaviour change

At the end of the intervention, participants received guidance on food reintroductions. This contrasts with other dietary interventions involving exclusions, in which the reintroduction phases were integrated into the study design,^(43)^ or were not applicable, such as in vegan or vegetarian trials. Most participants remained on the elimination phase for 4-8 weeks while selectively reintroducing foods, typically those they most missed, such as eggs, before discontinuing the formal process.

Although the AIP diet was widely considered too restrictive and mentally taxing for permanent adherence, most participants reported making meaningful, sustainable changes. These included retaining many AIP meals and preparation habits, becoming more mindful about food choices and identifying links between specific foods on symptom changes. These practices are consistent with behaviour change literature in weight management, where long-term success is associated with internalised motivation and a transition from external accountability to personal ownership.^(44,45)^

Dietary flexibility increased over time, with participants permitting occasional deviations in social settings. Similar patterns were reported in men undergoing dietary interventions during radiotherapy, who relaxed dietary rules in social contexts to avoid discomfort,^(33)^ and in a Swedish Mediterranean diet study in RA, participants also described relaxing dietary rules in some social settings while generally maintaining the diet over a five-year follow-up.^(12)^

In the PFJ study, follow-up 65 of 77 participants at 12 months found stable adherence and sustained clinical improvements compared to baseline.^(14)^ Key enablers of long-term adherence included self-monitoring, social support and an emphasis on consistency over perfection,^(46)^ factors echoed by AIP participants who adopted greater flexibility while maintaining core dietary practices. Ongoing social support also remained important for AIP participants, who continued to share family meals with minor personal modifications, and in social situations, hosts often accommodated dietary preferences. This contrasts with the Swedish Mediterranean study with five-year follow-up, where some participants reported resistance from family and partners who refused to eat the same meals.^(12)^

Self-efficacy varied among individuals. While most maintained partial adherence, one participant was unable to maintain dietary restraint, citing lack of motivation and discipline. This reflects findings from a Swiss study, where low adherence was associated with emotional eating, ambivalence toward consuming palatable foods and reduced physical activity, whereas successful long-term adherence was correlated with higher dietary quality and regular exercise.^(47)^ Psychological traits, such as a higher internal locus of control, may also influence both participation and long-term success. In a 13-month vegetarian diet trial for RA, participants scored significantly higher on this trait and lower on the “chance” subscale compared to non-participants, suggesting a belief in personal control over health and openness to non-conventional therapies.^(48)^

### Implications for practice and future dietary interventions

Successful implementation of restrictive dietary interventions in RA may be supported by providing clear education, practical resources, professional guidance, encouraging household involvement and support, and opportunities for peer support and connection. Attention should be paid to the psychological and practical demands of dietary transition, and to providing structured support for food reintroductions and long term flexibility and sustainability. While remote delivery appears feasible, adverse effects may be more readily identified through in-person contact.

## Strengths and limitations

This is one of the few studies to qualitatively examine the enablers and challenges experienced by people with RA during and after a dietary intervention. It is the only known study to conduct interviews with participants both immediately post-intervention, and one year later without follow-up support, providing insight into the real-world experiences of implementing and sustaining the highly restrictive AIP diet.

All participants were interviewed twice, providing a breadth of perspectives, including that of an AIP dietary non-responder. While the study included two males (22%), which adds value given the female predominance in RA studies, the sample size was small and self-selected via social media. Like participants in the MedRA and PFJ studies, they demonstrated high motivation and may not be representative of the broader RA population. Findings may be most transferable to adults with RA who are motivated to explore dietary approaches and are willing to undertake substantial dietary change. Experiences may differ among individuals with lower motivation, greater socioeconomic constraints, limited cooking skills, or less social support.

The interviewer was also the lead researcher and a registered nutritionist who delivered the intervention. While this could introduce social desirability bias, participants nonetheless provided candid feedback and constructive suggestions, indicating a reasonable level of openness.

## Conclusion

Participant motivation to trial the AIP diet was primarily driven by curiosity about the effect on RA symptoms, with additional influences including altruism and the appeal of a disease-specific dietary approach. While the initial mental and physical demands of the intervention were high, adherence was strongly supported by household involvement, social support, and guidance from the researcher. Upon conclusion of the study, most participants did not follow the reintroduction protocol in a structured way, often abandoning it in favour of a more flexible approach. Nevertheless, many retained foundational dietary habits and described increased awareness of how food affected their RA symptoms. Key factors contributing to long-term adherence included internalised motivation, peer and household involvement, and the ability to adapt the diet sustainably to everyday life. Future interventions should consider these elements to enhance feasibility, adherence, and real-world applicability.

## Supporting information

10.1017/jns.2026.10135.sm001 McNeill et al. supplementary material McNeill et al. supplementary material

## Supplementary material

The supplementary material for this article can be found at https://doi.org/10.1017/jns.2026.10135.
