# Supplementary material for: Exploring participant experiences with the autoimmune protocol (AIP) diet in rheumatoid arthritis: a longitudinal qualitative study
Source: J Nutr Sci. 2026 Aug 3;15:e73. doi: 10.1017/jns.2026.10135 (PMC13439018; doi:10.1017/jns.2026.10135)
Supplement: Supplementary file 1 [file S2048679026101359sup001.docx]

**Supplementary information:**

**Exploring participant experiences with the autoimmune protocol (AIP) diet in rheumatoid arthritis: A longitudinal qualitative study**

**Julianne McNeill, Gael Mearns, Rebecca Grainger, Caryn Zinn**

Contents

[Interview Questions	1](#_Toc234589800)

[Table 1: Interview 1, semi-structured interview questions, immediately post intervention	1](#_Toc234589801)

[Table 2: Interview 2, semi-structured interview one-year post intervention	2](#_Toc234589802)

[Themes and subthemes, immediate post study	2](#_Toc234589803)

[Table 3: Post-study, Theme 1	2](#_Toc234589804)

[Table 4: Post-study, Theme 2	3](#_Toc234589805)

[Table 5: Post-study, Theme 3	7](#_Toc234589806)

[Themes and subthemes, one-year follow-up interviews	8](#_Toc234589807)

[Table 6: One-year post-study, Theme 1	8](#_Toc234589808)

[Table 7: One year post-study, Theme 2	10](#_Toc234589809)

[Table 8: One year post-study, Theme 3	12](#_Toc234589810)

# Interview Questions

## Table 1: Interview 1, semi-structured interview questions, immediately post intervention

Table 1 Indicative questions for semi-structured interview post 8-week AIP intervention

1. What was your view of the AIP diet prior to starting it?
2. What was your motivation to participate in the study? Was there anything specifically that made you interested?
3. Describe how you went about starting AIP
4. Can you tell me about any challenges or difficulties you had changing to the AIP diet?
5. What things helped you start on AIP?
6. What was your experience of the ease or difficulty in locating and buying allowed food over the 8 weeks?
7. In what way did your spending pattern change on the AIP diet compared to your usual diet?
8. What was your experience of the ease or difficulty sticking to the diet for 8 weeks?
9. What was your experience of following the diet in social settings, at work, other people’s places, eating out or at restaurants?
10. How supportive were the people in your life for you taking on and staying on this eating plan?
11. How do you rate the diet on taste and palatability? What did you dislike food wise, what did you like?
12. What was your experience of feeling satisfied or hungry on the diet?
13. Did you have cravings, or was there food you missed?
14. What did you discover about the way foods or diet affect you? Mentally, physically
15. In what ways did AIP affect your RA?
16. Did you notice any changes in your health (better or worse) on this programme (not related to RA symptoms or questionnaires)?
17. What are your thoughts about your diet going forward?
18. Is there anything you would suggest for people in future studies using AIP?
19. What was your experience of support for you on this programme from researchers?
20. Is there anything you would like to add about your experience of being on this diet and taking part in the study?

## **Table 2**: Interview 2, semi-structured interview one-year post intervention

Table 2 Indicative interview questions on one year post intervention.

1. Tell me about what happened with your diet once the study ended.
2. How has your diet evolved over the last year?
3. What was your experience of the food re-introduction protocol? Tell me how you went about that.
4. Did you observe any reactions to foods reintroduced?
5. In what ways has your diet changed as result of the participating in the study?
6. How would you describe your current diet?
7. Tell me about any challenges or difficulties you have managing your diet:
   - Locating or buying food
   - Eating outside the home
   - Travel and holidays
8. Have there been things that have enabled you to stay on your current diet?
9. How supportive were the people in your life for staying on your current diet?
10. How has your RA been over the last year since the study ended?
11. What did you discover about the way foods or diet affect you? Mentally, physically?
12. Going forward. What are your thoughts about your diet?
13. Looking back to the end of the study, is there anything that would have been beneficial to have, for example information or support?

# Themes and subthemes, immediate post study

## ****Table 3:** Post-study, Theme 1**

| **Theme 1: Open to change** | | |
| --- | --- | --- |
| **Subtheme** | **Quotes** | **Code** |
| **Motivation to change** | P1: “But I'd had a note to sort of look into it (AIP) and find out more about it. So then when I saw your post, I thought, well, this is the perfect opportunity”  “I guess, it just aligned with my philosophies and beliefs anyway.”  P2: “There was no motivation, really for anything else except to benefit myself to get me on the track to get a decent diet going… I just wanted a cleaner diet”  P7, F: “So tried not to go in with any expectations except for just to get my eating back on, in control which when I've done before and maybe not to this amount - has kept the flare ups at bay,”  P4: “Because I know it's so critical for people like that to learn on on, you know, actual humans…If it makes a difference to other people, then that's awesome. Yeah, it's good to be involved in something like this.” | Responding to timely opportunity  Aligned with philosophy  Gain or regain control of health and manage symptoms  Contribution to others and science |
| **Trust in guidance and protocol** | P1: #1 “what I've discovered from reading books about it and stuff is, there are quite a few different ideas. Like some of them, say, to cut out meat completely, um, so there are slight variations of it. So it was good to sort of do it with, with guidance as to, you know, what you should and shouldn't do and what else you could try and that sort of thing”.  P2: “It's so good to have a qualified dietician to guide you and to push you on the right track and to help you through whereas when you're on your own. You just don't have, I didn't know what to do.”  P2: “Every diet that I've ever looked at before has been a weight loss diet. This is more about me, it's more about my health… And this is a completely different diet, it’s a diet about autoimmune. It's about your immune system. It's about getting yourself better.”  P7: “So it was really good at explaining why, you know, I shouldn't have peanuts because you know the legumes and the nuts and the seeds, and why, why are autoimmune disease guts bad about like, because again, the research I did just learning about the gut barrier, and the immune barrier right next to it, and you'd learn how and why it's so important what you eat, and to keep that gut healthy, so nothing crosses over…I think it's just because the AIP diet is specified for autoimmune. So it's not a diabetic diet. It's not your cardiac diet. It's for me,” | Clarity of advice  Qualified guidance  Confusing advice around diet  A diet specifically for autoimmune, not general, not weight loss  Scientific rationale is logical |
| **Curiosity and hope about a different approach** | P11: “I wouldn't say any expectations. I was just, you know, really open minded … I was just really curious to know if it would work or have, you know, any effects… just really intrigued to see whether, you know, diet would help with pain and inflammation.”  #4, F: “Well, my initial thoughts were if this was something that could help me decrease my pain meds for RA, then, you know, it was something that I thought was exciting to try… I didn't have a lot of expectation, because I had kind of tried various different types of healthy eating and what have you, and that didn't work.”  P5: : “oh, just a little bit of hope, really, you know, not not expectations, I wasn't going in thinking you know, how, how great it was going to be or, or how it might affect us,” | Curious and open-minded, not expectation  Excited to try  Hopeful new approach might help RA  Hope not expectation |

## ****Table 4:** Post-study, Theme 2**

| **Theme 2: Navigating dietary transition** | | | | |  |
| --- | --- | --- | --- | --- | --- |
| **Subthemes** | | **Quotes** | **codes** | |  |
| **Working around realities of diet transition** | | P5: “probably more concerned about my stomach and whether I was going to be eating enough”… “there's a lot of things that disappear. You know, I'm gonna love to come home and have a beer and a cracker and a bit of cheese and stuff like that. And that was all gone, you know and bacon and eggs on the weekend, and all that all the stuff that, you know, we kind of look forward to. it was, it seemed like was disappearing,” “And I kind of immediately went into panic mode and thought there wasn't too much left”  P6: “in the four that first four weeks, I ate a lot of things that I knew I wasn't going to be allowed (laugh)…(concern about) missing out on things, initially nuts and seeds because when I opened the pantry it's like all these jars of nuts and things… It wasn't hard, there was there was certainly there was some emotional reluctance”  P11: “when I actually saw the yes and no list and you know, how strict it was? Yeah, I wasn't sure if I was going to be able to achieve it. You know, especially the odd glass of wine or alcohol.” | Concerns – deprivation, hunger  Anticipatory overeating  Emotional reluctance  Doubt in ability to adhere | |  |
|  |  | P10: “the four weeks before we started the, I guess there's a bit of preparation in there of psyching yourself that you're not going to be allowed to have these foods in four weeks time”  P2: “And yet the whole concept of just a flat out cold turkey on not just one or two, but absolutely everything. It was quite daunting to start off with. And that's where the mental came in as I had to actually apply myself to say this is this is 100% commitment.”  P7: “ I did a lot of research because I wanted to know what I was in for…. I think I did pretty good in the four weeks leading up to doing the AIP diet so I wasn't caught off guard too much.” | Cognitive priming  Psyching self up  100% adherence commitment  Pro-active information seeking | |  |
|  |  | P4, Solo parent: “I didn't want to have to think about what I wanted to eat or come up with recipes. I've got a busy lifestyle and that to me would just be adding stress…I just went basic… just cooking that basic food you know my veggies and the meat and just eliminated all the herbs that I couldn't have”  P5, Female partner: “I had a whole folder with four weeks of meals kind of planned” P5: J… had put this plan together I could see food on it- breakfast, lunch and dinner so it was kind of, took away that panic a little bit”  P2, F: “I had a look at all the information you sent us. I had an extensive, I printed out wads and wads and wads of recipes, I had a look at what was required, I had a look at what I wanted to eat and what I enjoy eating and I extracted what I needed from that… I went online and had a look and I also went and hit the shops… and then the Sunday I spent all day cooking and I was in the kitchen all day preparing”  P7: “I did a lot of grocery shopping stocking up beforehand mentally to get make sure that I was never caught without food”  P6, F “I put everything that we couldn't use away somewhere else.”  P1: “I went through the kitchen with a red marker and I put red ticks on stuff, and told everyone these are the things that that I can have, so if you're making something, you know, this is what you can add for me and not other things” | Personalised preparation strategy:  Minimalist approach  Extensive planning  Stocking and pre-preparing  Environmental organisation  Removing non-compliant foods  Marking non allowed foods | |  |
|  |  | P11: “it was completely foreign to begin with. And, you know, it's like, oh, you know, just thinking about what, what am I going to cook, you know, looking up recipes and all that”  P7, F: “the awareness of checking everything that you're eating, 'What am I doing?' 'oh my goodness this is I can't do eggs for breakfast, okay we're gonna try this for breakfast, no didn't like that for breakfast.” | Cognitively demanding | |  |
|  |  | P5 partner, F: “So you basically go from cooking one meal a day to three meals a day so yeah, there was definitely preparation there.”  P2: “I had to make my own bread, I had to make my own pancakes, I had to make everything. …I had to cook and prepare everything myself so the time in the kitchen virtually quadrupled I was in the kitchen all day for the first week.” | Time-intensive  From convenience to make from scratch | |  |
|  |  | P5: “Yeah I think we kind of, our both of our systems had sort of took a while to adjust, but that was probably a week or something like that.. Bit of constipation in the household  P7: “two days of not bad headaches…just feeling like crud the first couple of days”  P2: “when I first went on for diet I was quite moody and quite ratty because I couldn't have the sweet and I couldn't have the glass of wine and I couldn't have the chocolate I was, you sort of get quite agitated | Adaptation symptoms  Physical and emotional discomfort  Effects of withdrawal | |  |
| **Subtheme** | **Quotes** | | **Codes** | |  |
| **Managing / dealing with challenges to sustain adherence** | P4: “just prepped, prepped on the weekend getting all the vegetables down and what have you, it was pretty much it…I've kept it simple- two reasons cost and easier to think about.”  P11: “I've had this thermo mix, it's been absolutely brilliant. Doing lots of AIP soups which yeah, have for breakfast as well, and, or midafternoon if I'm hungry.”  P11: definitely more time consuming with preparation. Because, you know, we, we are a busy household. Yeah, things have slowed down over lockdown. So, you know, it definitely helped. | | Weekend prep, simple food  Batch cooking  Lockdown benefit, more time | |  |
|  | P11: “I think my go to for sugar craving I'll have you a few dates. … I found these apple crisps, apple chips. Absolutely love them.”  P2: “and something that did help was olives. I just had an olive or three and it took away the cravings.”  P4: “Well you did say I could eat dark chocolate… that has satisfied me enough not to feel that I need some extra sweets … I can survive on a couple of pieces now whereas before it might be a whole block.”  P11: “I was cutting up cheese for their burgers last night and all I wanted to do was eat a piece of cheese, my mouth was watering” JM “What stopped you eating it?” P11 “thinking that might have a negative effect”  P5: “you have those moments where you think…icecream will be nice ... and if we weren't on the study, then I'm damn sure I would have had a couple.  P10: Some of the food wasn't as enjoyable, like the cassava flour and the tortilla. You know, we'd try and make pancakes and we didn't like the flour in it. I think we tried pancakes. And they were just horrible, they were disgusting. We tried to disguise them with raspberries and all sorts but we just couldn't eat them.  P6: I think there's a lot of spices and things that I missed.  P11: “I think one of the biggest thing was not having a coffee in the morning… you know, the first week or so it was really quite hard… but I managed.”  P3: “(I missed) the eggs and beans I think because it was that easy breakfast that I used to have.. just convenient…and filling me up more”  P7: “I think there's always that little thought in the back of my head because I see a bag of chips and I'm like Oh, I do miss the taste of those and I smell the coffee and I Oh, I miss the taste of that. But the willpower to not do it wins. Yeah, so there's always that little thought but the gains and all the goodness are stronger.” | | Managing sweet cravings  Getting past temptations  Fear of negative effect  Commitment to study  Recipe failures  Unpalatable food  Bland food  Missing spices  Missing coffee  “I managed”  Missing familiar breakfast  Using willpower  Focus on positive benefits | |  |
|  | P1: “I would have to probably contact the cafe and say 'Can you do this?' 'Can you modify this?' and you know, I would have felt like I was being a bit of a hassle… So, I think if we hadn't gone into lockdown, I either would have not gone, or I probably would have just gone and ordered a herb tea.”  P11: “we've had outdoor things with our neighbors over the last few weeks. And I've just taken along my own dinner and my soda water every time.”  P6F: “we went to their place, they had fish and she prepared one separately for me … so she knew about it she was perfectly happy to cater for me yeah” | | Social situations: requesting = discomfort  Take own food and drink  Host support | |  |
|  | P1: So six, sort of five week five or week six. the novelty I guess had worn off. And it just felt like there was still quite a long way to go.  P6: “I knew it was a fixed period of time. And there was in some, at some points in the middle I was kind of like counting down.” | | Diet fatigue  Fixed timepoint helped | |  |
|  | P10: a bit more to start ‘cause we were stocking up on all these new things and coconut flowers and cassava flowers and um and then it just steadied off. And I would say it's just about the same really.  P6: I didn't do much in the way of alternative stuff like I didn't do any baking things  P11: It's not like one go-to place to get all your products sort of thing. And then the shipping costs as well, I think I got one order from Albany or something that was like $16 for shipping. | | Initial cost higher, stabilised  Managed cost by avoiding baking  Multiple online shops if buying specialty items | |  |
| **Subtheme** | **Quotes** | | | **Codes** | |
| **Enablers of adherence** | P7: “I’m on a clinical trial. I need to follow this. I wanted to do it as best as I could, because I don't like letting people down, and you don't get the full benefits if you don't do it correctly.”  P11: “having somebody there like, you know yourself. Who's been checking in and reporting to as such every week. It's like, I can't cheat! I don't know. It's all mindset, I think. …I'd feel really guilty if I had something.” | | | External accountability  Sense of obligation  Potential benefits of doing it properly  Guilt from deviation | |
|  | P1: I'd cook it for a few days. So one day, I'd have to cook it and the next day, I might just have to heat it up  P5 partner: So the first sort of four weeks, I kind of stuck to the plan. Just because it was there. And it was easy. It was almost like having a food box. It's like I don't have to think about what I'm going to cook I'm just gonna cook what's on my plan and then and but also we often eat a bit like that anyway you know, a bit of a tray bake like you do with you know the chicken and all the mixed veggies all on one tray in the oven” | | | Batch cooking  Planning reduced cognitive load  Simplified cooking | |
|  | P11: “definitely my husband doing it as well…I just think having somebody eat the same foods as you doesn't make you feel so isolated.”  P1: sometimes other people in the family would cook the recipe and I would just say can you take a bit of this out…before you add the spice mix.”  P4: “They [young adult children] were pretty supportive, like, if I went to grab something, say if they were having fries and I went to grab a fry off their plate, they were "Mum, don't don't do it"…  P5: “So we'd say ‘Oh, would you like a gin?’ Which meant the soda. (laugh) ‘Oh, yes, I'd love a gin thank you’ (laugh)”  P1: “having teenagers in the house…they'd sort of make a joke. Like, they'd be eating, say, hamburgers or something. And I'd be like, ‘How are they?’ And they're like, ‘Oh, no, they're awful, Mum. You wouldn't want any.’ (laugh)”  P3: “I couldn't have done it if K… [wife] wasn't helping me with it.” | | | Shared meals reduce isolation  Family involvement in cooking  Verbal encouragement  Humour as support  Gendered support roles, females supported males | |
|  | P2: “And the Facebook page has been absolutely brilliant. Yeah, actually, that's been also very beneficial as a Facebook page following other people and knowing that there are other people out there who going through the same thing as you are. I'm not on my own.”  P1: Facebook group was a little bit useful, but probably would have worked better if everyone could have possibly met first, even though it's a small group, I didn't really know those people… someone posted on there that they were making nomato sauce and I was like, OH, and so then I made that that, you know, became Bolognaise and all that sort of thing. So that, in that regard, it was really good.  P10: I wasn't on Facebook, which probably didn't help because that's where people were coming together and, and sharing their stories and everything so I probably missed out on that. | | | Peer connection  Shared experience/community  Better connection if met first  Recipe sharing  Not on FB, missed connection | |
|  | P3, wife: “It was excellent just having you being at the end of an email, you always responded really quickly. It was brilliant. And everything was so clear. The recipes were amazing. It was SO well done, such a good trial.”  P4: “those lists were awesome to have. They were up and my fridge throughout.”  P1: what would we have done without Google? You know, if we tried to do this 10 years ago, would have been different, because having the ability to just Google a recipe or look up AIP, something or other, you know, that was really good. | | | Researcher support Responsive communication  Clear dietary guidance  Resources  Online resources accessed | |
|  | P11F: “it became, you know, it became easier as time went on. And yeah, it's, it's definitely becoming more normal now. Whereas it was completely foreign to begin with… where as now I don't really think about it. It's just, this is the way of eating”  P2: “Now I know all the recipes I don't have to keep looking at the book and that sort of thing but it was a lot less time consuming.” | | | Habit formation  Dietary behaviour automation  Familiarity over time | |
|  | P11F “previously because I was, I say was, a really, a real sweet tooth as well, like, Yeah, I like chocolate. I'm just like, really bad with chocolate…. we're already getting chocolate treats put in our trays at work and all that, and, but no I haven't been tempted to open them just hid them away. Which for me, that's a huge thing.”  P10: “I loove cheese and crackers, you know, like, you know, you drink, and even a drink of gin.. So within a week or two, I didn't miss it at all”  P7: “I wasn't going hungry. Right so the meals that I would eat I don't weigh things I've told you that before I just ate until I don't want it and sometimes a little bit more.. but I never felt I was missing stuff. So feeling full after breakfast until lunchtime came and then not you know, seeing everybody else is surrounded by so many treats at work and I didn't reach for them… I ate what I wanted and I lost weight … I just didn't feel deprived”  P3; wife: we'd never go back to normal lasagna now. I made a butternut lasagna. Yeah, it's delicious. And we wouldn't stop eating that now.  Those chicken patties I'd make again and with the grated apple and the chicken the ones that came on the Facebook page someone had made Yeah, they were yum!. P3: The nomato sauce was really good too | | | Decreased cravings  Increased satiety  Enjoyment of new recipes | |

## ****Table 5:** Post-study, Theme 3**

| **Theme 3: Reflecting on benefits and looking ahead** | | |
| --- | --- | --- |
| **Subtheme** | **Quotes** | **Codes** |
| **Perceived health changes** | P11: “virtually no joint pain in my hands now which is fantastic…I am aware of pain in one foot but it's nowhere near as bad as what it used to be like, I’d feel that within 3 Ks sort of thing. But now I can easily walk you know, 8, 9 Ks…there's definitely less inflammation than I've EVER had.”  P7: “I was definitely able to sleep better, I usually wake up with pain and have to stretch a certain way. There's not the pain. So, I haven't been having to do that.”  P4: “a lot more energy. Didn't feel tired in the middle of the day.” | Reduced pain and inflammation  Increased exercise tolerance  Improved sleep, less pain  Increased energy |
|  | P4: “I had really bad indigestion for probably about six weeks before the study… I had to sit up in the middle of the night kind of thing rather than lie down. So that that totally disappeared.”  P7: “I would go through constipation and diarrhoea before the AIP whereas now it's like every morning clockwork regular”  P7: “my skin is glowing, … I've always had adult acne which is really bad and wouldn't go away - cleared up.”  P6: “I have a lot less headaches. That was the big difference.”  P2: “I was having hot flashes, probably three, four times a day. I think if I've had four hot flashes since I've been on the diet” | Resolution of indigestion  Improved bowel regularity  Clearer skin  Reduced headache frequency  Fewer hot flashes |
|  | P3: “it didn't change anything with my arthritis.” “My guts hasn't been right. For the last probably four or five weeks”  P10: “I've lost too much weight... I'm not hungry. I don't have an appetite. I'm tired. …“Energy level's definitely down. Sjogren’s is the same pretty much” on the other hand: “I have noticed is my digestion is definitely better… bloated tummy and rumbling and gas, have reduced by about 80%.” | Negative gastrointestinal impact  Increased RA symptoms  Better gut |
| **Future dietary intentions** | P4: “I'd like to keep as much of the way I'm eating now. As part of my life, daily life, at the same time, I don't want to restrict myself if I am going out for dinner or what have you. I don't want to say no, I can't have that.” | Modified flexible approach |
|  | P7: “I've never felt better as a human so why would I destroy that? …I'm going to continue …another four weeks or eight weeks longer or so before even start reintroducing foods.” | Continue strict AIP |
|  | P2: “Why mess it all up? … where the real mental attitude comes in now is the maintaining. Because it's so easy to say, well, I've done my bit for eight weeks, and I know I feel good. Of course, as soon as you feel good, you start stuffing yourself full of rubbish again.” | Self-doubt in ongoing adherence |

# Themes and subthemes, one-year follow-up interviews

## ****Table 6:** One-year post-study, Theme 1**

| **Theme 1: Personalised re-introductions and responses** | | |
| --- | --- | --- |
| **Subtheme** | **Quotes** | **Codes** |
| **Approaches to reintroductions** | P5: “We were quite structured…we'd do a group at a time, so kind of, you know, kind of nuts and maybe some cereal. And when we've done a couple of weeks on that, we probably did about six weeks reasonably conscientiously bringing groups in and with no sort of real adverse effects.”  P4: I didn't have it as structured as I was in the structure. I didn't worry too much about things like the dressings and sauces, and what have you. I was just more mindful of the types of food I was eating, and nightshades and that kind of stuff.  P7: I kept going for, I think it was three, three and a half months in total on the strict AIP. … I was feeling quite good, and then slowly started reintroducing some things that I really missed… I once again got it from the Dr. Sarah Ballantyne's book. And every time I do try and reintroduce something I bring it out to remind myself what her instructions are.  P11: “I think mostly with eggs because I really, that was a big thing for me was eggs, and just reintroduce them slowly and yeah, I was okay. And coffee was the other thing. Which Yeah, I think yeah, I've been okay.”  P1: My peanut butter toast came back first, because it's what I missed the most. [laugh] So I still most mornings have gluten free bread with the peanut butter on it. Yeah, I didn't notice. Like I didn't notice any adverse reactions or anything. | Structured reintroduction by food groups  Informal reintroductions  Extended strict AIP  Structured as per Ballantyne  Desired foods reintroduced first |
|  | P2: “after about three weeks, it became quite sort of, I documented everything, it started becoming quite boring. So I would start on other stuff example potatoes, which I knew I reacted very badly to. And I had a bad reaction but then I'd just carry on … So probably lack of discipline, particularly stupid to say the least....It was almost like, the diets done, I've lost all the weight, I'm feeling fantastic. Here we go. Let's just go wild….And within a month after the three month mark. I was in a really bad way. And I started picking up the weight, it was more the weight that worried me than how I felt. Which is crazy.”  P10:” No, it was something I couldn't do, I think I started, I did two days of, I think I did milk or something like that. And then my family was like, “This is ridiculous. Just eat!” So I pretty much stopped it and tried just to eat just whatever I could.” | Reintroductions got boring  Reversion to old habits  Chose not to do reintroductions  Weight regain prioritised |
| **Responses to reintroduced foods**. | P2: “Joint ache, headache, within 10 minutes of having a potato, a white potato I had my joints were aching all over, headache and it took about a day before it settled down… Tomato I ache constantly all my joints ache, my hands are very bad… the areas where my rheumatoid affected me I had a very bad reaction to nightshades they're the killers.” “Gluten, I very badly reacted to,… upset stomach, headache and joint, very bad joints….my irritable bowel kicked in within 10 minutes. And it is incredibly uncomfortable. It's terrible cramps, stomach cramps, upset stomach”  P6: “potatoes….it'll be sometime in the next 24 hours. I'll just have like, generally my toes will go sort of one of the toes will start zinging like quite an electrical pain. And it might last at the most kind of four or five minutes and it's pretty unpleasant but it's not, you know, not enough put me off curly fries.” I'm pretty clear that if I went back to having nightshades at the level that I was, I would have a higher level of pain.”  P11: we went out for a meal, and it was the first time I'd had a curry, and my joints were sore. Like I think because when we went to bed and then I woke up the next day and I was really quite sore.  P4: if I eat a lot of dairy, it doesn't agree with me. So I'm on the toilet quite a bit. It's that I haven't seen a rise in inflammation from eating too much dairy… Yeah. really sore stomach and diarrhea.  P6: Gluten: More digestive ill effect rather than joints. I haven't specifically put joint pain relative to gluten. No, but But yeah, I know. I feel gluggy is my term for it. If I do have a lot of it.  P11: bread, bread I know straightaway, sore gut, quite literally within an hour. Yeah. Sore gut like bowel changes.  P5: we probably did about six weeks reasonably conscientiously bringing groups in and with no sort of real adverse effects.  We'll have a bit of alcohol, we'll have some chocolate, we'll have some, we'll have some coffee. Those sorts of things in our lives and don't seem to be, don't think they're impacting. | Clear negative reactions  Nightshades  Gluten  Potato  Spicy restaurant meal  Dairy - digestive effects, not inflammation  Dairy -GI effects  Bread – GI effects  No noticeable effects from some reintroductions |

## ****Table 7:** One-year post-study, Theme 2**

| **Theme 2: Finding and sustaining balance** | | |
| --- | --- | --- |
| **Subtheme** | **Quotes** | **Codes** |
| **Seeking balance** | P4: “October, middle of November, I was pretty much staying to the diet. But once that period ended, and I started getting into Christmas, that diet, everything went out the window…. Because I found being under the diet doing the diet was really restrictive. And it was, like taking up a lot of my brain matter”.  P5: the AIP was really, it was quite, you know, there was lots of preparation, lots of work to stick at it.  P5: “So just on the spectrum, we're kind of we're not down the AIP AIP end, but we're certainly not back at we were. I don't know. We're probably somewhere in the middle, you know, pretty considered and concious about what we eat. We'll have a bit of alcohol, we'll have some chocolate, we'll have some coffee. Those sorts of things in our lives don't seem to be, don't think they're impacting | Cognitive load too high  Physical demands unsustainable  Modified AIP |
| **Retaining AIP-inspired habits** | P4: “I do have a lot more anti- inflammatory food products in my diet… a protein shake with the blueberries and beetroot and stuff like that. That's a regular thing for me now…breakfast… I'm finding during the week, my protein shake fills me up to a point where I'm not hungry enough to eat lunch.  P11: “I'm pretty good going to work. Like, I'll make a salad. In the mornings, I'll cook up steak or cook up a piece of, you know, chicken thigh or breast or whatever, slice it up, put it in, still having coconut aminos for dressings…because the easy option would be to make a sandwich or make a bread roll, but I won't. And yeah, that's one thing I've stuck to”  P5: “J… (partner) is a really conscientious cook, so the veggies, big on veggies and meats and all that sort of thing. So the (AIP) diet's pretty much stayed. And now we've gone to probably an 80:20 gluten free.”  P3: The diet pretty much went back….maybe a little bit less of the bad stuff maybe.. Bread, we never really got back to bread either … Fish has gone up a bit more  P1: Like, it kind of like reminded me about eating more variety,…before it I got complacent and we just have white rice all the time. But now I make sure we have black rice and brown rice and white rice and different grains.  P5: So there's, you know, there's a lot more, I guess, consideration now that we kind of understand a bit more. So, I think that was a big part of it, as well, there's a lot of a lot of education, you know, in terms of in terms of what's good and what's bad. And, and, and, yeah, looking for signs, you know, what's what's, what's working. What's what's, what's keeping (kicking) this off. Way more conscious of that. Yeah, definitely. Yeah. Yeah, that's probably, that's probably been the biggest thing really, is that decision that you sort of go through when, you know, when you're looking at what you should and shouldn't have. | Improved food quality  Satiety  Home prepared lunches maintained  Continued vegetable intake  Less bad stuff, more fish  More plant variety  Conscious of food effects  Considered choices |
|  | P3W: We haven't really gone back to much rice.. or any pastas. Bread, we never really got back to bread either .. we don't eat so much cheese now.  P4: I guess I've pretty much eliminated dairy from my diet. But that's probably it. I'm mindful about how much gluten I eat, but not to the point where I don't have it.  P5: Veggie wise, not to, you know, we're careful with nightshades…maintenance seems to be staying away from gluten as much as I can  P6:“I'm pretty clear that if I went back to having nightshades at the level that I was, I would have a higher level of pain  P11: Still mostly dairy free. Don't eat a lot of bread. Like sugar, not as much as before. (Alcohol) Not really, as much as, definitely nowhere near as what we were before. | Gluten avoidance  Reduced dairy intake  Nightshade moderation  Dairy, bread, sugar and alcohol reduction |
|  | P5: “we've gone back to chocolate and coffee and things like that. So they're probably stuff that we shouldn't have.”  P6: “I've probably got a little bit more inclined to have a little bit of something that I shouldn't but become more relaxed, but you know, it will still only be once a week max type thing.” | Moral framing of food affecting decisions |
|  | P7: So I've been very close to strict AIP with just a couple of reintroductions since in that year since coming off. | Maintained strict AIP with a few reintroductions  A choice for health |
|  | P2: I wasn't working with you anymore, I was on the diet, but I wasn't working with you any longer. So the challenge was, am I going to stay on the diet and look after myself or absolutely deviate and go bananas and I went bananas, there was no challenge. I didn't have any fight within myself at all. I just went sort of okay, I'm not on the diet, and well, let's go. And that was it.  P2: I'm basically off the diet. I'm not in any way strict or restricting myself to the diet. | Unable to sustain dietary habits  Full reversion without study structure |
| **Subtheme** | **Quotes** | **Codes** |
| **Maintaining everyday habits** | P11: But my husband and I both will try and eat healthily during the week and try and modify what what we're having without, you know, having completely different meals, say than the kids  P7: I meal prep every weekend religiously, whether it's Saturday or Sunday or a mixture of both. | At home, shared meals, eat healthily  Meal prep continues |
|  | P6: if I'm out somewhere, if people ask me, Is there anything you don't want? I'll say, no nightshades if possible, but if I'm served food with nightshades on it's, it's an occasional thing. | Managing eating at others homes |
|  | P5: And most places do have like, most places will have gluten free options. And I mean, most places are pretty accommodating now with low dairy and all that sort of thing.  P1: if I do go out, I just look for the best option of what's on the menu… You know, there might be an ingredient in it that wouldn't have been allowed on the AIP, but that doesn't bother me anymore.” | Eating out – choices aligned with preferences  Flexible re food not usually eaten |
|  | P6: “We went on a boat trip recently. Six days. And I said to them, gluten free and stay off the tomatoes. Yeah. And that was fine. They catered for that.”  P10: “Staying with family. I took quite a bit of food with me so I took all my snacks. my own porridge and basically took all my own breakfast stuff. And then dinner was always pretty good anyway.”  P7: “we're going to Melbourne a friend and I. We booked an apartment hotel so we've got a full-fledged kitchen available to us, and I'm gonna go to the markets and there's a grocery store nearby, and we're going to do that mostly.” | Travel strategies: requests of hosts  Taking own food for travel  Self-catering accommodation |
|  | P6: “we've got a wedding coming up where the food is going to be Italian. But it's a one off.”  “But if I'm out somewhere, if people ask me, ‘Is there anything you don't want?’ I'll say, ‘no nightshades, if possible’. But if I'm served food with nightshades on it's, it's an occasional thing.”  P5: “Yeah, we try to sort of, we're not monks, that's for sure. You know, so we still like to go out and do things, if there's a decent option on there, and there often is now, we'll look for that… for me now, the maintenance seems to be staying away from gluten as much as I can. It feels like a good balance.” “We'll have a bit of alcohol, we'll have some chocolate, we'll have some, we'll have some coffee. Those sorts of things in our lives and don't seem to be, don't think they're impacting.” | Social eating flexibility  Flexible mindset  Balanced approach |

## ****Table 8:** One-year post-study, Theme 3**

| **Theme 3: Reflections on wellbeing and future intentions** | | |  |
| --- | --- | --- | --- |
| **Subtheme** | **Quotes** | **Codes** |  |
| **Sustained symptom reduction** | P1: I'd say it's been really good. I haven't had too many flare ups. … Yeah, it's pretty good. Manageable. … I still just take the methotrexate and the folic acid  P4: since the study, I think my arthritis has definitely been managed, where I'm not having huge flare ups or anything, though, it's been pretty consistent that way…. I have ongoing appointments with the specialist every three or four months, and they're pretty happy that it's stabilised. And compared to last year, the inflammation they did another scan of my wrist where I was having a lot of issues. There was hardly any inflammation there this year  P5: I think that study was kind of a shifting point for me…. But I mean, I'm certainly way, way, way way better than I was, you know, where I am from, y'know, pretty, pretty bad place three years ago, three or four years ago. And, and that's been a progression. And in the last couple of years, I mean, you know, I mean, it's worlds apart, you know… I mean I feel terrific. You know, that's really good…. I really don't get swelling, it's sort of like, it's hard because I can't I, I sort of don't know whether it's the drugs or the diet or a combination of both you know? Yeah. But I'm feeling pretty good, best, best I have in years really.  P6: I've had very little pain this year. Yeah, you know because what I was getting was like at night I'd get my toes would start zinging and things like that but I've had hardly had any of that… I'm probably more pain free. Since I first had rheumatoid actually.  P7: It's been so good. I almost forget I have it. Because I have not had a flare up, It's been, that's been amazing. So yeah, you almost just forget except for you got really ugly knee joints | Stabilised RA over the year  Reduced inflammation  Intervention a turning point  Very little pain  No flare-ups |  |
| **General wellbeing changes** | P5: “I mean I feel terrific…, I feel tip top. I'm going to be really happy if I can maintain this, y'know?”  P7: “Sleep, sleep is so much better. Massively. .. I used to have a lot of insomnia, troubles falling asleep, staying asleep”  P11: “I'm good actually, not bloated like how it used to be. I used to be quite bloated all the time.”  P4: (indigestion that stopped with AIP?) “no, I haven't had any of that. I haven't had any issues with that at all.”  P7: I've just gotten to the point where I don't want to go back to that pain that I had. I feel I feel like a human again. …I'm actually like everybody else and the fact that I can just train again. … So seeing all those results that just keeps me going. | Feel terrific  Better sleep  Not bloated  Indigestion stayed away  Can train like others |  |
|  | P2: So I'm basically back where I was a year ago, I'm feeling absolutely awful. My joints are aching, I have headaches, my irritable bowel has gone bananas, I have a sinus problem. My eyes are gritty, I'm having hot flushes again…. And now I'm back to exactly where I was a year ago. So I'm not feeling particularly bright. So to answer your question, no, I'm not doing very good…. I started putting on weight. It's taken me a year to put on 10 of the 13 I lost. I've lost my energy level. I'm sluggish, I am constantly aching. I have a backache, my stomach's a mess. So everything's just got my health has just got progressively worse and worse and worse since I stopped the diet. | **Outlier: complete reversion** |  |
|  | P11: I'd say my energy levels have probably dropped again. You know, because I felt like I had so much energy while I was doing the diet. | Less energy than on AIP |  |
| **Persistent health challenges in AIP diet non-responders** | P3: I don't know if you remember, but towards the end of the diet, my stomach wasn't very good. So and that's still sort of going on now. We've got some new probiotics now that seems to be helping a bit. But it's yes, it just hasn't been quite right the whole time. we did the colonoscopy and the did the endoscopy, we did everything.  P3: I was just feeling tighter in my joints but it wasn't painful. Just that it, yeah, just tighter. Not moving as freely maybe. And when he scanned it, he said, there's quite a bit of fluid in them.  P10: in May, I went to a nutritionist, because I really hadn't gained weight. So I've been with her for six months, I'm pretty much I put my weight back on. And I'm back on track. I wasn't able to exercise probably when I started with her, because I was just too sore and too painful. I'm feeling a bit better as that, so I'm, I'm doing some weights and walking…it's just been a slow, slow progress. Yeah. It's probably only the last, out of that six months, the last month that my energy levels have come up like the last week or two. So basically, I've only put on muscle. Yeah, very little fat | Gut issue not resolved  Joints have more fluid, stiff  RA activity – sore, painful  Nutritionist input for weight regain |  |
| **Future dietary intentions** | P2:“I'm gonna make a good go of it again. But it's been very difficult mentally in the last last couple of months, in the last year, acknowledging and realising exactly what damage I've done.”  P4:“I know that I'm going to make changes… having salads on a nightly basis or like the roast veggies, making those up again for the week,… getting rid of my wheat not having breads.. making things from scratch… but keeping that simple as well.”… I'd like to say 100% I'd keep out wheat, gluten and gluten foods, that's what I'd like to do.  P11: I'd say my energy levels have probably dropped again. You know, because I felt like I had so much energy while I was doing the diet. | Back on AIP for wellbeing  Improve quality, avoid wheat  Desire to restore energy | |
|  | P1: “try and sort of keep with the same philosophy as it were, keep with the philosophy of eating as much variety as I can. As much fresh fruit and veg, try and keep with the variety of vegetables… I feel quite well and quite happy. I wouldn't want to cut out those things that I do enjoy, like my peanut butter toast.”  P5: I think it's lifestyle now, where we are is lifestyle, that's not going to change. | Continue with plant variety  Maintain dietary enjoyment  Diet is lifestyle | |
